# Improving Parkinson's Disease Care through Systematic Screening for Depression

**DOI:** 10.1002/mdc3.14163

**Published:** 2024-07-19

**Authors:** Connie Marras, Zachary Meyer, Hongliang Liu, Sheng Luo, Sneha Mantri, Allison Allen, Sydney Baybayan, James C. Beck, Amy E. Brown, Francis Cheung, Nabila Dahodwala, Thomas L. Davis, Megan Engeland, Conor Fearon, Nicole Jones, Kelly Mills, Janis M. Miyasaki, Anna Naito, Marilyn Neault, Eugene C. Nelson, Ebubechukwu Onyinanya, Carlos Ropa, Daniel Weintraub

**Affiliations:** ^1^ The Edmond J. Safra Program in Parkinson's Disease and the Morton and Gloria Shulman Movement Disorders Center, Toronto Western Hospital, Department of Medicine, University of Toronto Toronto Ontario Canada; ^2^ Parkinson's Foundation New York New York USA; ^3^ Department of Biostatistics and Bioinformatics Duke University Durham North Carolina USA; ^4^ Department of Neurology Duke University School of Medicine Durham North Carolina USA; ^5^ Department of Neurology The Parkinson's Disease and Movement Disorders Center, Johns Hopkins University Baltimore Maryland USA; ^6^ Department of Neurology Vanderbilt University Medical Center Nashville Tennessee USA; ^7^ Division of Neurology, Department of Medicine University of Alberta Edmonton Alberta Canada; ^8^ Department of Neurology University of Pennsylvania School of Medicine Philadelphia Pennsylvania USA; ^9^ Department of Community and Family Medicine at Geisel School of Medicine at Dartmouth College The Dartmouth Institute for Health Policy and Clinical Practice Lebanon New Hampshire USA; ^10^ Departments of Psychiatry and Neurology University of Pennsylvania School of Medicine Philadelphia Pennsylvania USA; ^11^ Department of Psychiatry Parkinson's Disease Research, Education and Clinical Center, Philadelphia Veterans Affairs Medical Center Philadelphia Pennsylvania USA

**Keywords:** depression, screening, mental health, Parkinson's disease

## Abstract

**Background:**

Depression is common in Parkinson's disease (PD) but is underrecognized clinically. Although systematic screening is a recommended strategy to improve depression recognition in primary care practice, it has not been widely used in PD care.

**Methods:**

The 15‐item Geriatric Depression Scale (GDS‐15) was implemented at 5 movement disorders clinics to screen PD patients. Sites developed processes suited to their clinical workflow. Qualitative interviews with clinicians and patients provided information on feasibility, acceptability, and perceived utility.

**Results:**

Prior to implementation, depression screening was recorded in 12% using a formal instrument; 64% were screened informally by clinical interview, and no screening was recorded in 24%. Of 1406 patients seen for follow‐up care during the implementation period, 88% were screened, 59% using the GDS‐15 (self‐administered in 51% and interviewer administered in 8%), a nearly 5‐fold increase in formal screening. Lack of clinician or staff time and inability to provide the GDS‐15 to the patient ahead of the visit were the most commonly cited reasons for lack of screening using the GDS‐15; 378 (45%) patients completing the GDS‐15 screened positive for depression, and 137 were enrolled for a 12‐month prospective follow‐up. Mean GDS‐15 scores improved from 8.8 to 7.0 (*P* < 0.0001) and the 39‐item Parkinson's Disease Questionnaire emotional subscore from 42.2 to 36.7 (*P* = 0.0007).

**Conclusions:**

Depression screening in PD using a formal instrument can be achieved at much higher levels than is currently practiced, but there are barriers to implementing this in clinical practice. An individual site‐specific process is necessary to optimize screening rates.

Depression is common in people with Parkinson's disease (PD)^1^ and has a significant negative impact on quality of life.^2^ Within Parkinson's Foundation Centers of Excellence (PF COE) a substantial proportion of patients with symptoms of depression do not receive either pharmacologic intervention or other mental health services, such as counseling or psychotherapy.^3^, ^4^ This suggests that systematic screening for depression in patients with PD could identify untreated depression and hopefully trigger appropriate management, leading to improved quality of life. In the Parkinson's Foundation Parkinson's Outcome Project, a longitudinal cohort study enrolling patients with PD without exclusions for monitoring of PD treatments and outcomes,^5^ the use of mental health professionals (ie, psychiatrists, psychologists, or social workers) varies substantially among COEs, as does the proportion of patients prescribed antidepressants, suggesting that practice changes at the center level could improve care.

In primary care settings, routine screening using brief questionnaires is recommended as a strategy to improve depression recognition and care.^6^ The American Academy of Neurology includes annual assessment of mood disorders as a quality metric; however, it was still found that assessment rates for any psychiatric disorder (psychosis, depression, anxiety disorder, apathy, or impulse control disorder) were less than 40%.^7^, ^8^ Based on these observations, our project assessed the feasibility and preliminary effectiveness of systematic depression screening of PD patients attending PF COEs using a formal screening questionnaire and identified barriers to its routine implementation.

## Patients and Methods

Five tertiary care movement disorders centers in the United States and Canada participated. The study consisted of 4 parts.

### Retrospective Chart Review of Consecutive Patients

To document the frequency and methods of depression screening in the year prior to any intervention, the records of 10 consecutive patients per health‐care practitioner (HCP) having appointments for follow‐up PD care at each PF COE or virtually were reviewed for information related to depression screening, diagnosis, and treatment. The method of screening was categorized as either a clinical interview (ie, inquiry about depressive symptoms in an unstructured conversational style) or an administration of a structured questionnaire.

### Implementation of Depression Screening

The coordinating site developed a process map for implementing depression screening for both in‐person and virtual visits (see Supporting Information Figure S1) and shared these with sites, which developed their own process, tailored to their clinical operations. Resources were provided (see Supporting Information), including (1) patient‐facing educational resources (Figure S5); (2) a suggested depression treatment algorithm (Figure S2); and (3) the HOPE‐D (Healthcare Options for People Experiencing Depression), a depression‐specific shared decision‐making tool.^9^ Sites submitted weekly data on screening rates. At virtual monthly meetings, which included all site principal investigators and research coordinators, comparative trends in screening rates between centers and over time were reviewed, implementation challenges were discussed, and solutions were proposed.

The 15‐item Geriatric Depression Scale (GDS‐15) was chosen as the depression screening tool based on its good discriminant validity in PD,^10^ self‐report format, and relatively short administration time of <5 minutes.^10^, ^11^, ^12^ A short, patient‐facing written explanation of the purpose and rationale for screening for depressive symptoms accompanied the GDS‐15, which was self‐administered or interviewer administered, based on the HCP's preference. Further depression assessment and treatment were undertaken at the discretion of the HCP.

### Prospective Follow‐Up

Patients scoring ≥5 on the GDS‐15, which has been proposed a screening cutoff to detect clinically significant depressive symptoms,^10^ were invited to participate in follow‐up assessments. The 39‐item Parkinson's Disease Questionnaire (PDQ‐39, a PD‐specific health‐related quality‐of‐life instrument) was administered at the time of enrollment. The PDQ‐39 and GDS‐15 were administered again at 12 months.

### Key Informant Interviews with HCPs and Patients

Semistructured interviews were conducted with 2 HCPs and 2 patients from each site who had screened positive for depression on the GDS‐15. Interviews with patients explored the experience of depression and depression screening. HCPs were asked about the impact of screening on clinical workflow, challenges in implementation, and sustainability. For interview guides see the Supporting Information Figures S3 and S4. HCP interviews were completed by video conferencing; patients were interviewed by telephone with the optional assistance of a care partner. The interviews were recorded, and transcripts were reviewed to identify themes using grounded theory analysis and phenomenology.^13^ Once site processes were optimized, interviews were conducted with each site principal investigator, focusing on the implementation of depression screening. An electronic survey was also distributed to all clinicians at each site to obtain additional information regarding the utility and feasibility of the screening program.

### Analysis

Sample size was based primarily on the aim to increase the rate of screening for depression and secondarily to increase the rate of newly detecting depression. Assuming that the baseline rate of screening is 70%, we estimated 90% power to detect an increase to 80% with a sample size of 200. Assuming that the baseline rate of newly recognized depression is 10%, we would have 90% power to detect an increase in this rate to 20% with a sample size of 132. Thus, we aimed to observe screening practices from at least 200 patients attending follow‐up care at each site after implementation of GDS‐15 screening, from which we aimed to recruit 132 participants to prospective follow‐up.

Descriptive statistics were used to summarize the data: mean and standard deviation (SD) for continuous variables, and counts and percentages for categorical variables. Paired *t*‐tests were used to compare PDQ‐39 and GDS‐15 scores between baseline and the 12‐month follow‐up.

## Results

Three centers in the United States and 2 centers in Canada participated. The number of clinicians ranged from 3 to 9 at each site (median 6).

### Retrospective Review of Depression Screening

The medical records of 310 patients seen by 29 HCPs in the year prior to the implementation of systematic screening were reviewed (Table 1). Prior to the implementation of systematic GDS‐15 screening, an average of 76% (range: 56%–100%) of patients were screened. Only 12% (range: 0%–57%) were screened using a formal depression instrument; instruments used were the GDS‐15, Patient Health Questionnaire‐2 (PHQ‐2),^14^ Movement Disorder Society‐sponsored revision of the Unified Parkinson's Disease Rating Scale (Part 1 depression item),^15^ Nonmotor Symptoms Questionnaire,^16^ the Beck Depression Inventory,^17^ and the Beck Depression Inventory Fast Screen.^18^ At Toronto, records of 20 patients per clinician were sampled to investigate screening practices separately for visits conducted virtually and in person. At this site, 50% of patients (35 of 70) attending the clinic in person were screened for depression, compared with 63% of patients (44 of 70) whose visits were conducted virtually. All screening was informal by clinician interview.

**TABLE 1:** Depression screening practices in the year prior to introducing GDS‐15 screening, in a consecutive sample of patients attending each center for PD follow‐up care

Characteristics at baseline	Toronto Western Hospital, Toronto, Canada	Vanderbilt Medical Center, Nashville, USA	Johns Hopkins Hospital, Baltimore USA	University of Alberta Hospital, Edmonton, Canada	Pennsylvania Hospital, Pennsylvania, USA	All
Number of HCPs	7	4	4	3	6	29
Patient characteristics						
N	140	40	40	30	60	310
Age (mean, SD)	69.9 (10.6)	72.2 (8.8)	68.6 (9.3)	69.8 (10.1)	68.3 (9.3)	69.7 (9.9)
Sex (number of male, %)	79 (56.4)	23 (57.5)	25 (62.5)	18 (60.0)	38 (63.3)	183 (59.0)
Race (N, %)						
White	105 (75)	39 (97.5)	34 (85)	24 (80.0)	49 (81.7)	251 (81.0)
Black	1 (0.7)	0	2 (5)	0	4 (6.7)	7 (2.3)
Asian	18 (12.9)	1 (2.5)	2 (5)	2 (6.7)	0	23 (7.4)
Indigenous	0	0	0	1 (3.3)	0	1 (0.3)
Native Hawaiian/Pacific Islander	0	0	0	0	0	0
Other	4 (2.9)	0	2 (5)	0	7 (11.7)	13 (4.2)
Unknown	12 (8.6)	0	0	3 (10)	0	15 (4.8)
Ethnicity (N, %)						
Hispanic	4 (2.9)	0	1 (2.5)	0	0	5 (1.6)
Non‐Hispanic	37 (26.4)	40 (100)	39 (97.5)	27 (90)	58 (96.7)	201 (64.8)
Missing	99 (70.7)	0	0	3 (10)	2 (3.3)	104 (33.5)
Disease duration, years (mean, SD)	9.6 (6.0)	7.6 (5.2)	8.4 (8.0)	8.4 (6.7)	7.5 (5.8)	8.7 (6.2)
Pre‐implementation depression screening						
N screened (%)	79 (56.4)	40 (100)	40 (100)	21 (70)	55 (91.7)	235 (75.8)
Instrument(s) used (N)	0	0	PHQ‐2 (1) MDS‐UPDRS (1) BDI (2)	NMSQuest (12)^a^ GDS‐15 (3)^a^	UPDRS (2) BDI (5)^b^ BDI‐FS—7 (5)^b^ Neuro PD‐10 (12)	
Screened by clinical interview, N (%)	79 (100)	40 (100)	36 (90.0)	7 (33.3)	38 (69.1)	200 (85.1)
Both	0	0	4 (10)	2 (9.5)	11 (20)	17 (7.2)

^a^ One individual completed both the NMSQuest and the GDS‐15.

^b^ Five patients completed multiple screening instruments.

Abbreviations: GDS‐15, 15‐item Geriatric Depression Scale; PD, Parkinson's disease; HCP, health‐care practitioner; SD, standard deviation; PHQ‐2, Patient Health Questionnaire‐2; MDS‐UPDRS, Movement Disorder Society‐sponsored revision of the Unified Parkinson's Disease Rating Scale; BDI, Beck Depression Inventory; BDI‐FS, Beck Depression Inventory Fast Screen for Medical Patients; Neuro PD‐10, a site‐specific 10‐item interview guide for PD care; NMS Quest, Non‐motor Symptom Questionnaire.

### Screening Implementation

At 2 centers, the GDS‐15 was sent to patients prior to their appointments using a patient‐facing internet‐based portal linked to the electronic medical record (EMR) system. If the patient did not complete the questionnaire prior to the visit, paper forms were used as backup (1 center) or electronic presentation at a check‐in kiosk (1 center). At 2 centers, paper forms were provided to the patients upon arrival in clinic, to be completed as they waited to see their clinician. At 1 center (n = 107) the movement disorder neurologist administered the GDS‐15 to the patient by interview during the appointment, due to challenges sending the form by electronic means to a patient population with limited internet access and technical knowledge, and not having an efficient way to identify PD patients at check‐in for a workflow separate from non‐PD patients. Despite an emphasis on clinical implementation, 2 centers used research personnel, for tasks such as emailing GDS‐15 survey links to patients who did not have EMR access, inputting scores into the EMR, checking for completion of the GDS‐15 ahead of the visit, and approaching patients in the waiting room to complete the GDS‐15 if they had not already done so.

The implementation period varied across sites from 11 to 20 weeks (median: 17 weeks); 28% of patients seen during this period were seen virtually (range: 13%–54% across sites). From a consecutive sample of 1406 individuals, 1242 were screened for depression (88%, range: 74%–97% across sites), of whom 830 were screened using the GDS‐15 (59%). Screening rates in those seen virtually were 44%, compared with 56% for in‐person visits. During the last week of the implementation period, the proportion of patients screened using the GDS‐15 by site ranged from 50% to 71% (median: 54%). Visual inspection of the run charts over the implementation period suggested that increasing the number of clinicians participating in the screening program and increasing numbers of virtual visits were associated with subsequent reduction in screening rates (Supporting Information Figure S6).

Among the patient encounters where screening with the GDS‐15 did not occur, the most common HCP‐reported reasons were as follows: (1) patient had not completed the questionnaire in advance, and there was insufficient time in the waiting room or during the visit (n = 223, 38%); (2) inability of the patient to communicate by electronic means or lack of internet access (n = 162, 28%); and (3) insufficient time on the part of staff (n = 138, 24%). For 26 patients a language (4), cognitive (19), or physical health (3) barrier to completing the GDS‐15 was cited. Twenty patients declined to complete the questionnaire. Of note, during the virtual monthly meetings clinicians mentioned that they often would forget to review the completed GDS‐15 in the absence of reminders.

A total of 412 patients (29%) were screened using a method other than the GDS‐15, most commonly by informal clinician interview. The most commonly used formal instruments other than the GDS‐15 were the PD‐10 (a site‐specific 10‐item interview guide for PD care, in 28) and the PHQ‐2 (in 58), both at a single site. Compared with the entire consecutive sample, those screened were more likely to be White (*P* = 0.0002) but were not meaningfully different in age, sex, or disease duration from those not screened (Table 2).

**TABLE 2:** Screening rates achieved and comparison of those screened to not screened for depression from a consecutive sample of patients receiving follow‐up PD care

	Toronto Western Hospital, Toronto, Canada	Vanderbilt Medical Center, Nashville, USA	Johns Hopkins Hospital, Baltimore USA	University of Alberta Hospital, Edmonton, Canada	Pennsylvania Hospital, Pennsylvania, USA	All
All (N)	200	107	230	200	669	1406
Screened (any method) (N, %)	164 (82)	104 (97)	215 (93)	148 (74)	611 (91)	1242 (88)
Screening methods among those screened						
GDS‐15 (N, % of all seen)	107 (53.5)	55 (49.5)	164 (71.3)	135 (67.5)	369 (55.2)	830 (59.0)
Other (list N, % of all seen)	57 (28.5)	49 (45.8)	51 (22.2)	13 (6.5)	242 (36.2)	412 (29.3)
Reasons for not using GDS‐15 (N, %)						
Declined	8 (8.6)	0	1 (1.5)	0	9 (3.0)	18 (3.1)
Insufficient time in clinic	84 (90.3)	5 (9.6)	1 (1.5)	31 (47.7)	78 (26.0)	199 (34.6)
Other	1 (1.1)	47 (90.4)	28 (41.8)	34 (52.3)	185 (61.6)	295 (51.2)
Missing	0	0	36 (55.0)	0	28 (9.3)	64 (11.1)
Age (mean, SD)						
All	70.1 (9.7)	69.9 (9.1)	67.9 (9.3)	69.3 (10.0)	70.2 (9.1)	69.7 (9.3)
Screened^a^	69.0 (9.3)	70.8 (8.2)	68.2 (9.3)	68.9 (10.2)	69.8 (8.7)	69.3 (9.1)
Sex (N male, %)						
All	133 (66.5)	71 (67)	153 (66.5)	120 (60)	455 (62.4)	932 (63.6)
Screened^a^	74 (69.2)	40 (74.1)	107 (65.2)	74 (54.8)	230 (62.3)	525 (63.3)
Race (N, % White)						
All						
Screened^a^	86 (43)	107 (100)	196 (85.2)	99 (49.5)	592 (81.2)	1080 (73.7)
Black	73 (68.2)	55 (100)	142 (86.6)	69 (51.1)	331 (89.7)	670 (80.7)
All						
Screened^a^	2 (1)	0	19 (8.3)	1 (0.5)	61 (8.4)	83 (5.7)
Asian	1 (0.9)	0	13 (7.9)	1 (0.7)	17 (4.6)	32 (3.9)
All						
Screened^a^	33 (16.5)	0	10 (4.3)	11 (5.5)	29 (4)	83 (5.7)
Other	19 (17.8)	0	6 (3.7)	8 (5.9)	12 (3.3)	45 (5.4)
All						
Screened^a^	0	0	0	2 (1)	1 (0.1)	3 (0.2)
Unknown	0	0	0	2 (1.5)	1 (0.3)	3 (0.4)
All	79 (39.5)	0	5 (2.2)	87 (43.5)	46 (6.3)	217 (14.8)
Screened^a^	14 (13.1)	0	3 (1.8)	55 (40.7)	8 (2.2)	80 (9.6)
Disease duration (mean, SD)						
All	8.7 (5.1)	8.7 (6.2)	9.0 (5.7)	10.2 (6.3)	7.4 (6.1)	8.3 (6.0)
Screened^a^	7.7 (4.6)	9.5 (6.2)	9.0 (5.9)	9.7 (6.3)	7.6 (6.0)	8.4 (5.9)

^a^ Screened using GDS‐15.

Abbreviations: PD, Parkinson's disease; GDS‐15, 15‐item Geriatric Depression Scale.

### Qualitative Interviews and Surveys

Ten HCPs (8 physicians, 2 nurse practitioners) completed semistructured interviews. A major theme emerging from the interviews was the value of a formal depression questionnaire as a reminder for the clinician to ask about mood symptoms. In many cases, HCPs felt the GDS‐15 expanded the agenda of the clinical visit: “It probes more deeply than a superficial single question. … I think sometimes it takes people off guard.” Three HCPs commented that having the GDS‐15 score was useful in reflecting back to the patient the degree of his or her depressive symptoms; in at least 1 case, the clinician felt that was instrumental in convincing the patient to agree to treatment; 94.4% of respondents agreed that the GDS‐15 was a useful screening measure for depression in people with PD, and 64.7% reported that the screening program led to a change in clinical management for at least 25% of participants. The majority (76.5%) recommended that the screening program should be implemented at other PF COEs.

Conversely, some HCPs raised concerns about time constraints in the clinical encounter, particularly when patients had multiple symptoms to address. One site principal investigator noted, “There's so many symptoms, and especially non‐motor symptoms that people could self‐report. And how do we balance the screens with just like, regular doctor patient interaction during the visit?” Specific challenges noted included the time burden on coordinators or other staff to ensure that patients had completed the screen prior to the clinical visit, as well as concerns that some of the GDS‐15 questions were ambiguous in distinguishing between motor and depressive symptoms, leading to additional discussion with the HCP for clarification. This was reflected in the survey as well, with 55.6% of respondents agreeing or strongly agreeing that “the depression screening process slowed the clinical workflow.” Additionally, technological barriers (eg, lack of patient access to messaging through the EMR) led some sites to rely on paper forms, which were often easier to administer and interpret but led to restrictions in screening capability for patients seen virtually during periods of lockdown.

Ten patients (age at diagnosis: 46–70 years, disease duration: 2–14 years, 4 women) completed semistructured interviews; no care partners participated. All patients felt that depression screening was important for comprehensive PD care, and several suggested the need for greater awareness of depression among people with PD (eg, through support group meetings, online resources). Eight of the 10 patients attributed their depressive symptoms to specific events (eg, death of a loved one, coronavirus‐related lockdowns).

Three patients noted that the wording of the GDS‐15 was confusing; 2 reported that they had completed it during the clinic visit and asked the clinician clarifying questions, and 1 indicated his or her care partner had completed the questionnaire. One participant questioned the value of the questionnaire: “I don't often get any sense of achievement or positiveness out of the exercise. I mean, I'll go through it. Apart from what I've just said, I do try to answer as truthfully as possible, which is not always easy. These questions don't necessarily have an answer.”

### Baseline Screening Results, Treatment Decisions, and Prospective Follow‐Up

A total of 378 of 830 patients screened using the GDS‐15 (45%) screened positive for depression, and 137 of these were enrolled in prospective follow‐up (Table 3). Depression screening methods in the prior year for this subgroup had been clinical interview in 32 patients, GDS‐15 in 2, other screening instruments in 13, and none in 90; 46 of 137 were thought to have a clinical diagnosis of depression according to the movement disorder HCP, 7 of whom were newly diagnosed. Sixteen individuals (12%) initiated new depression treatment, and 72 (52%) were already treated, of whom 19 (26%) had treatment adjusted after screening. Fifty (37%) remained untreated. The shared decision‐making tool was used in 69 individuals (50%). At baseline, mean GDS‐15 scores were 8.8 (SD 3.6). By 12 months these had decreased to 7.0 (SD: 3.4), *P* < 0.0001. The mean PDQ‐39 total score changed from 36.4 (SD = 15.2) to 34.5 (SD = 14.4), *P* = 0.254. The mean PDQ‐39 emotional subscore changed from 42.2 (SD = 19.3) to 36.7 (SD = 19.9), *P* = 0.0007.

**TABLE 3:** Characteristics of patients screening positive for depression, treatment recommendation, and baseline GDS‐15 and PDQ‐39 scores

Characteristics at baseline	Toronto Western Hospital (N = 31)	Vanderbilt (N = 31)	Johns Hopkins (N = 25)	Edmonton, Alberta (N = 20)	UPenn (N = 30)	All sites (N = 137)
Age (mean, SD)	70.68 (9.1)	71.65 (7.89)	67.76 (9.16)	67.7 (9.18)	71.9 (5.93)	70.2 (8.32)
Male sex (N, %)	16 (55)	23 (74)	16 (64)	15 (75)	16 (53)	87 (63)
Race						
White (N, %)	26 (86.7)	31 (100)	24 (96)	17 (85)	30 (100)	128 (94.1)
Black (N, %)	0	0	0	0	0	0
Asian (N, %)	4 (13.3)	0 (0)	0 (0)	2 (10)	0 (0)	6 (4.4)
Other (N, %)	0 (0)	0 (0)	1 (4)	1 (5)	0 (0)	2 (1.5)
Hoehn and Yahr stage (N, %)						
1	4 (12.9)	0 (0)	0 (100)	0 (0)	0 (0)	4 (3.1)
2	9 (29.0)	5 (16.1)	9 (36.0)	4 (30.8)	19 (63.3)	46 (35.4)
2.5	0 (0)	0	6 (24.0)	7 (53.8)	2 (6.7)	15 (11.5)
3	9 (29.0)	15 (48.4)	7 (28.0)	1 (7.7)	9 (30)	41 (31.5)
4	7 (22.6)	8 (25.8)	3 (12.0)	1 (7.7)	0 (0)	19 (14.6)
5	2 (6.5)	3 (9.7)	0 (0)	0 (0)	0 (0)	5 (3.9)
Disease duration (mean, SD)	7.9 (4.17)	6 (4.67)	10 (5.67)	11.2 (8.15)	8.26 (4.69)	8.42 (5.60)
GDS‐15 administration method (N, %)						
Self	31 (100)	0 (0)	23 (92)	3 (15)	27 (90)	84 (61.3)
Interviewer	0 (0)	31 (100)	2 (8)	17 (85)	3 (10)	53 (38.7)
Clinical diagnosis of depression (N, %)	7 (22.6)	30 (96.8)	16 (64)	15 (75)	23 (76.7)	91 (66.4)
Newly identified depression (N, %)	0 (0)	2 (6.5)	0	4 (20)	1 (3.3)	7 (5.1)
Use of shared decision‐making tool (N, %)	3 (9.7)	31 (100)	1 (4)	7 (35)	27 (90)	69 (50.4)
Treatment recommended (new or ongoing) (N, %)						
New	1 (6.3)	5 (31.3)	4 (25.0)	3 (18.7)	3 (18.7)	16 (17.8)
Ongoing	7 (9.5)	23 (31.1)	17 (23.0)	8 (10.8)	19 (25.6)	74 (82.2)
GDS‐15 (N, mean, SD) baseline	7.68 (2.93)	12.58 (3.89)	8.08 (2.72)	9.05 (3.36)	7.43 (2.7)	9.01 (3.7)
PDQ‐39 (mean, SD) baseline	37.5 (14.8)	39.9 (14.1)	39.9 (16.8)	35.8 (17.2)	29.4 (12.5)	36.5 (15.3)
PDQ‐39 emotional subscale (mean, SD) baseline	44.0 (17.7)	43.7 (18.4)	45.0 (23.5)	41.0 (19.2)	41.1 (19.2)	43.0 (19.3)

Abbreviations: GDS‐15, 15‐item Geriatric Depression Scale; PDQ‐39, 39‐item Parkinson's Disease Questionnaire; SD, standard deviation.

## Discussion

During the implementation phase of our study to increase formal screening rates for depression in PD, only 59% of patients attending visits for follow‐up care for PD were screened using the GDS‐15 despite active efforts to promote screening and use of research personnel to accomplish it. However, this is nearly 5‐fold higher than the pre‐implementation formal screening rate of 12%. Our experience with implementing systematic screening with a formal questionnaire for depression provides useful observations which have implications for practice, expanding implementation and future research.

### Low Rates of Formal Depression Screening Prior to Implementation

Our retrospective review of consecutive visits prior to implementation demonstrates that formal screening using an instrument with known psychometric properties is uncommon in tertiary care movement disorders centers. Informal screening in the course of a clinical interview was the more common approach. Our experience is mirrored in an interview study with multiple sclerosis (MS) nurse specialists and neurologists.^19^ Most clinicians did not use depression screening instruments but preferred to decide who needed screening and to use their own questions. Reasons cited were lack of time or skills to formally screen for, or diagnose, depression. A few participating neurologists or nurse practitioners prescribed and monitored antidepressants, because they saw patients infrequently or perceived a lack of mental health management expertise. Similar concerns were found in a study of HCPs caring for patients with epilepsy.^20^ Lack of support staff and limited treatment options were found to be contributors to low screening rates in another study of MS.^21^

Given the high rates of informal depression screening occurring in movement disorders practices, it is important to question the added value of formal screening, but evidence is currently lacking in PD. A recent review concluded that there is insufficient evidence to recommend routine formal screening for depression in MS.^22^ The authors concluded that even assuming better detection, logistical, and resource requirements needs to be carefully considered. They further suggested that ultra‐short screens such as the PHQ‐2 (ie, asking about depressed mood and lack of motivation/anhedonia) may be a practical alternative to lengthier instruments. Acknowledging these practical considerations, and the certain variability in methods of informal screening, formal depression screening is still a standard to be aimed for if resources permit. Formal depression screening instruments have good performance in PD,^10^ can provide a measure of severity, and can track changes over time, and some can align with a DSM‐5‐TR (*Diagnostic and Statistical Manual of Mental Disorders*, fifth edition, text revision) diagnosis of depression. Furthermore, the use of a formal scale aligns with the call for measurement‐based care, which has been shown to improve patient outcomes in many settings, including mental health care.^23^

### Barriers to Implementation of Formal Depression Screening

White individuals were significantly more likely to be screened than non‐White individuals. Potential language and cultural barriers to screening will need to be investigated to promote health equity. Rates of screening with the GDS‐15 were lower for virtual than in‐person visits. The in‐person interaction provided an opportunity for several centers to ensure that those individuals who did not complete the GDS‐15 prior to arriving at the clinic completed it, which may account for the difference. Methods for overcoming this barrier are also needed.

In interviews with our participating HCPs, formal depression screening was felt to be useful but difficult to incorporate given all the potential clinical issues to be addressed at a given visit. This is despite the self‐report format of the GDS‐15. Our interviews with HCPs and patients revealed that follow‐up questions may be necessary to interpret the screening score. Given these findings, a shorter screening instrument may help to address the time barrier, although this remains to be tested.

Among 200 patients with various movement disorders (66% PD), the Emotional Thermometer, consisting of 4 or 7 visual analogue scales rating different emotions, durations, and burdens, and a short 7‐item screening instrument for depression designed for epilepsy were found to have acceptable accuracy for detecting depression.^24^ These were evaluated due to the perceived need for a screen requiring minimal time to complete and found that brief instruments can have acceptable sensitivity. Ultra‐short screening instruments for depression have been tested in epilepsy, stroke, and MS care as alternatives to longer instruments.^25^, ^26^, ^27^, ^28^, ^29^, ^30^ Ease of scoring or the lack of need for scoring altogether and greater acceptability for a broader range of patients with respect to cognitive and language abilities are cited advantages. Although experience has been mostly positive with such instruments, low sensitivity in some studies has been reported.^25^, ^27^, ^30^

### Personnel Required to Optimize Screening Rates

Even in a setting specializing in care of people with PD, depression screening with a formal instrument was difficult to achieve at high rates without the help of additional personnel beyond those assigned to clinical work. Personnel were required at some sites to ensure that the patient received the screening instrument (whether on paper or via an EMR) and that the results are conveyed to the clinician in time for the clinical encounter. The use of research personnel in our study likely artificially raised the screening rates and emphasizes the need for either dedication of additional clinical personnel to this effort or the use of electronic completion, scoring, and entry into the medical record to achieve comprehensive care.

### The Need for Site‐Specific Implementation Methods

The process for implementing depression screening was different at each site, indicating that fixed recommendations for how to incorporate formal depression screening into practice are unlikely to see satisfactory uptake. Guidance for clinics will need to be tailored to their unique characteristics. Relevant clinic characteristics include mechanisms for communicating with patients prior to their appointment, mechanisms for communicating results to clinicians, check‐in processes, and the frequency of virtual care.

### Potential for Improving Depressive Symptoms and Quality of Life

Nearly half of patients screened positive for depression, supporting the importance of screening for this issue in PD care. In a sample of those with GDS‐15 scores falling in the depressed range, we observed reductions in GDS‐15 and PDQ‐39 emotional subscores over 12 months. This is despite the fact that a low proportion (23%) of those not being treated at baseline chose to begin treatment. In the absence of an unscreened control group, it is not possible to conclude that screening influenced these scores; however, it raises the possibility that the depression screening process and associated discussions may have a positive impact on depressive symptoms. Notably, 8 of 10 patients who participated in the qualitative interview felt that their positive screen was due to an adjustment reaction or disorder rather than symptoms of a major depressive or persistent depressive disorder, and only one third of the prospectively followed sample were felt to have clinical depression in the opinion of the movement disorders clinician. These views may explain why a minority of screen‐positive patients pursued treatment. Formal assessments for a depression diagnosis were not mandated as part of this study, and the actual proportion of true positive screens cannot be determined. Nonetheless, these findings may reflect the known effect of stigma (actual or perceived) toward individuals with a mental health diagnosis, reducing the likelihood that affected individuals will pursue treatment.^31^ Challenges accessing mental health specialist care may also contribute, reducing the enthusiasm of practitioners for initiating or referring for treatment. Regardless of the explanation(s) for the low proportion of screen‐positive individuals initiating treatment, measurement‐based serial monitoring is appropriate to avoid the consequences of an untreated mood syndrome.

### Limitations

This experience in 5 centers likely does not demonstrate the full spectrum of contextual factors that can influence implementation. Building on this work, a larger implementation effort is necessary to learn more about the barriers and facilitators of depression screening in different settings. This includes collecting data on health system characteristics that may offer opportunities or present barriers to implementation. Ongoing follow‐up will be required to assess the sustainability of formal screening and how this screening impacts outcomes. In addition, results cannot be assumed to apply to nonacademic and nonmovement disorders practice. In the next phase of implementation, curtailing the use of research personnel would provide a more realistic estimation of the clinical resources required to accomplish widespread depression screening. Finally, the lack of prospective observation prior to implementation of the GDS‐15 or a control group for comparison prevents us from estimating the added value of this screening over usual practice.

## Conclusions and Next Steps

We have demonstrated that formal screening for depression in PD can be achieved at much higher levels than is currently practiced but that there are barriers to implementing formal depression screening in tertiary care movement disorders practice. A context‐tailored, site‐specific process is necessary to optimize screening rates. Alternatives to formal screening, or a choice of shorter formal instruments, may have greater uptake by HCPs. Given that informal screening is common practice, investigating the relative effectiveness of informal compared with formal screening would be relevant. This experience provides strategies that can be applied to a larger group of tertiary care movement disorder centers from which we can develop guidance for implementation processes that can be disseminated.

## Author Roles

(1) Research project: A. Conception, B. Organization, C. Execution; (2) Statistical analysis: A. Design, B. Execution, C. Review and critique; (3) Manuscript preparation: A. Writing of the first draft, B. Review and critique.

C.M.: 1A, 1B, 1C, 3A

Z.M.: 1C, 2B, 3B

H.L.: 2A, 2B, 3B

S.L.: 2A, 2B, 3B

S.M.: 1A, 1C, 3B

A.A.: 1B, 3B

S.B.: 1C, 3B

J.C.B.: 1A, 3B

A.E.B.: 1B, 1C, 3B

F.C.: 1C, 3B

N.D.: 1B, 1C, 3B

T.L.D.: 1B, 1C, 3B

M.E.: 1C, 3B

C.F.: 1A, 1B, 1C, 3B

N.J.: 1C, 3B

K.M.: 1B, 1C, 3B

J.M.M.: 1B, 1C, 3B

A.N.: 1B, 3B

M.N.: 1A, 3B

E.C.N.: 1A, 3B

E.O.: 1C, 3B

C.R.: 1C, 3B

D.W.: 1A, 1B, 1C, 3B

## Disclosures

**Ethical Compliance Statement:** This study was approved by the ethics committees of each of the 5 participating sites. Written informed consent was obtained from the individuals participating in the prospective follow‐up portion of the study and those participating in the qualitative interviews. A waiver of the requirement for consent was obtained from the ethics committees for the collection of data related to the retrospective medical record review and the baseline depression screening; these aspects of the work did not involve the disclosure of any individual‐level data outside the site providing care. We confirm that we have read the journal's position on issues involved in ethical publication and affirm that this work is consistent with those guidelines.

**Funding Sources and Conflicts of Interest:** Connie Marras, Daniel Weintraub, Eugene C. Nelson, Marilyn Neault, Sheng Luo, Hongliang Liu, Thomas L. Davis, and Nabila Dahodwala received grants or honoraria from the Parkinson's Foundation to conduct the study.

**Financial Disclosures for the Previous 12 Months:** Connie Marras has received honoraria from the Parkinson's Foundation and grants from The Michael J. Fox Foundation, the Parkinson's Foundation (United States), the International Parkinson and Movement Disorders Society, the Weston Brain Institute, and the U.S. National Institutes of Health. Zachary Meyer and Anna Naito were employed by the Parkinson's Foundation. Daniel Weintraub has received research funding or support from The Michael J. Fox Foundation for Parkinson's Research, the Alzheimer's Therapeutic Research Initiative (ATRI), the Alzheimer's Disease Cooperative Study (ADCS), the International Parkinson and Movement Disorder Society (IPMDS), the National Institutes of Health (NIH), and the U.S. Department of Veterans Affairs; honoraria for consultancy from Alkahest, Aptinyx, Boehringer Ingelheim, Cerevel Therapeutics, CHDI Foundation, CuraSen, Ferring, Medscape, Roche, Sage, Scion, Signant Health, and Takeda; and license fee payments from the University of Pennsylvania for the QUIP and QUIP‐RS. Allison Allen received honoraria from the Parkinson's Foundation, research funding from the Parkinson's Foundation, and salary support from the Huntington's Disease Society of America. Amy E. Brown served on the advisory board of Orphalan and has received income as investigator or sub‐investigator from Teva Pharmaceutical, CHDI Foundation, Alterity Therapeutics, Sage Therapeutics, Hoffman‐La Roche, Neurocrine Biosciences, PTC Therapeutics, Novartis, AbbVie, and UniQure. Thomas L. Davis has received income for consultancy from AbbVie Pharmaceutical and research funding from the Parkinson's Foundation. Nabila Dahodwala has received income for consultancy from Genentech and Mediflix; speaker honoraria from the World Parkinson Congress; research grants from the NIH, MJFF, the Parkinson's Foundation; and expert testimony fees from O'Brien and Ryan, LLP. Eugene C. Nelson received speaker honoraria from Planetree, MetroHealth (Cleveland), and the University of Texas Southwestern; research grants from the Crohn's & Colitis Foundation and NIHR‐UK; research contract fees from Pacific Source; and book royalties from Jossey‐Bass. Sheng Luo received consulting and advisory board membership with honoraria from the National Institutes of Health, the Parkinson Study Group, and IQVIA; research grants from the National Institutes of Health, the International Parkinson and Movement Disorder Society, and the Parkinson's Foundation; and salary support from Duke University. Sneha Mantri served as a consultant to Clinical Care Therapeutics and Modality AI; received honoraria from the Parkinson's Foundation and the Parkinson Study Group; received research grants from The Michael J. Fox Foundation and The Duke Endowment; and received grants as a site principal investigator for clinical trials sponsored by Neuraly, Biogen, IQVIA, and Takeda. Conor Fearon received salary support from the Edmond J. Safra Foundation and research funding from The Michael J. Fox Foundation. James C. Beck received research funding from the National Institutes of Health and was employed by the Parkinson's Foundation. Kelly Mills was a consultant for Tilosia Inc and the Parkinson's Foundation, and received research grants from the National Institutes of Health, The Michael J. Fox Foundation, and the Parkinson's Foundation. Marilyn Neault received consulting honoraria from the Parkinson's Foundation. Janis M. Miyasaki received honoraria from the Oxford University Press, the American Academy of Neurology, and the Parkinson's Foundation and research grants from PCORI, Brain Canada, the Canadian Consortium for Neurodegeneration in Aging, and the Social Sciences and Health Research Council. Megan Engeland, Hongliang Liu, Sydney Baybayan, Francis Cheung, Nicole Jones, Ebubechukwu Onyinanya, and Carlos Ropa have no disclosures.

## Supporting information


**Figure S1.** Process maps for Toronto Western Hospital (lead site). Process maps for the depression screening workflow developed for the Toronto Western Hospital. A similar map was developed by each of the sites based on this example but tailored to their own clinic structure and processes.
**Figure S2.** Treatment algorithm and suggestions. Suggested treatment algorithm and treatment suggestions provided to all sites.
**Figure S3.** Semistructured interview guides. Interview guide for interviews with patients and health‐care providers.
**Figure S4.** Feasibility and acceptability questionnaires of systematic depression screening and barriers to screening. Feasibility and acceptability questionnaires administered to health‐care providers and prospectively followed patients.
**Figure S5.** Patient‐facing educational materials. Patient‐facing educational materials provided to sites, for use at their discretion.
**Figure S6.** Screen percentage by dates of each center. Charts of percentage of PD (Parkinson's disease) patients screened over the implementation period used to monitor screening rates and process changes at each site. (A) Vanderbilt University, (B) University of Pennsylvania, (C) Johns Hopkins University, (D) Toronto Western Hospital, and (E) University of Alberta.
