# Supplementary material for: Improving Parkinson's Disease Care through Systematic Screening for Depression
Source: Mov Disord Clin Pract. 2024 Jul 19;11(10):1212–22. doi: 10.1002/mdc3.14163 (PMC11489616; doi:10.1002/mdc3.14163)

Figure S1:

Process maps for Toronto Western Hospital (lead site)

In-person visits

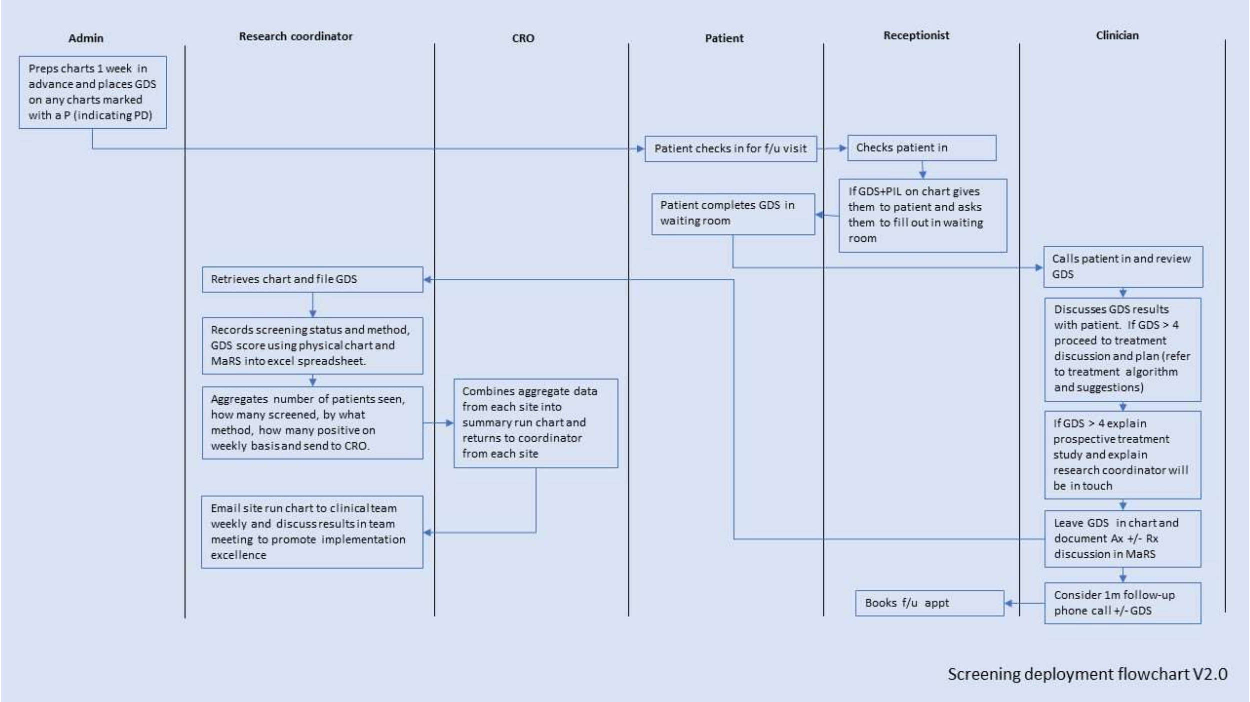

Virtual visits

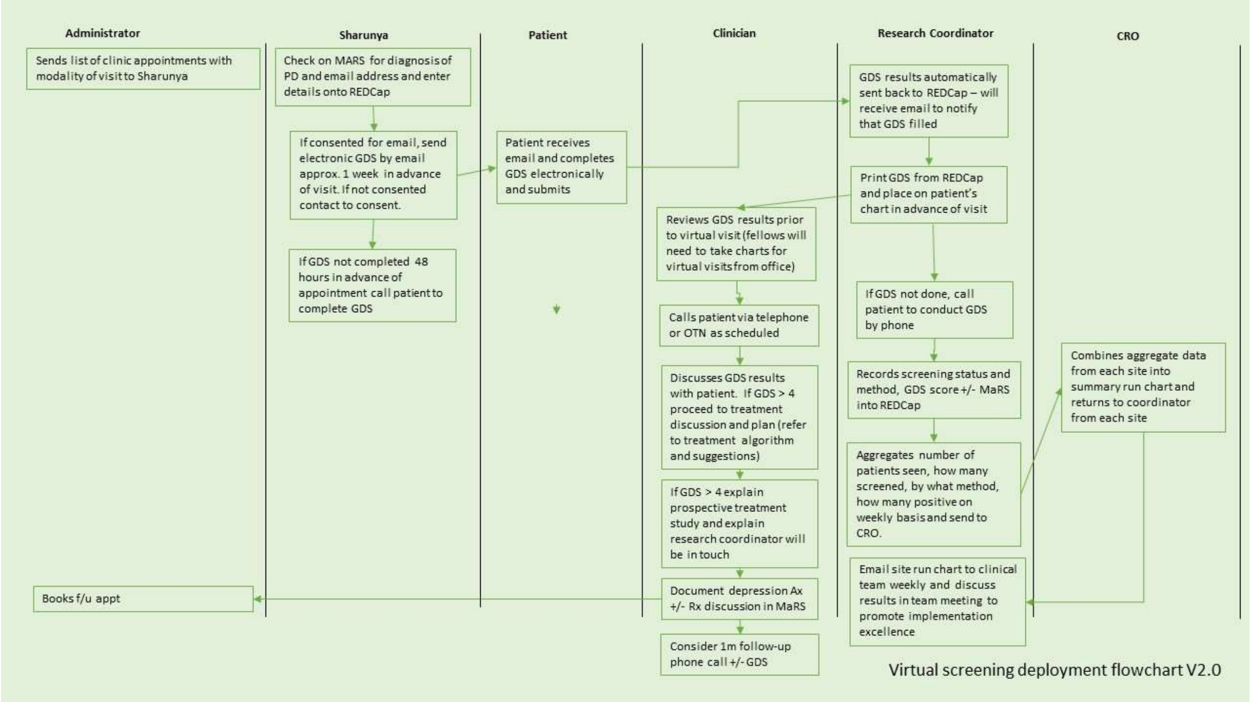

Figure S2:

Treatment algorithm and suggestions

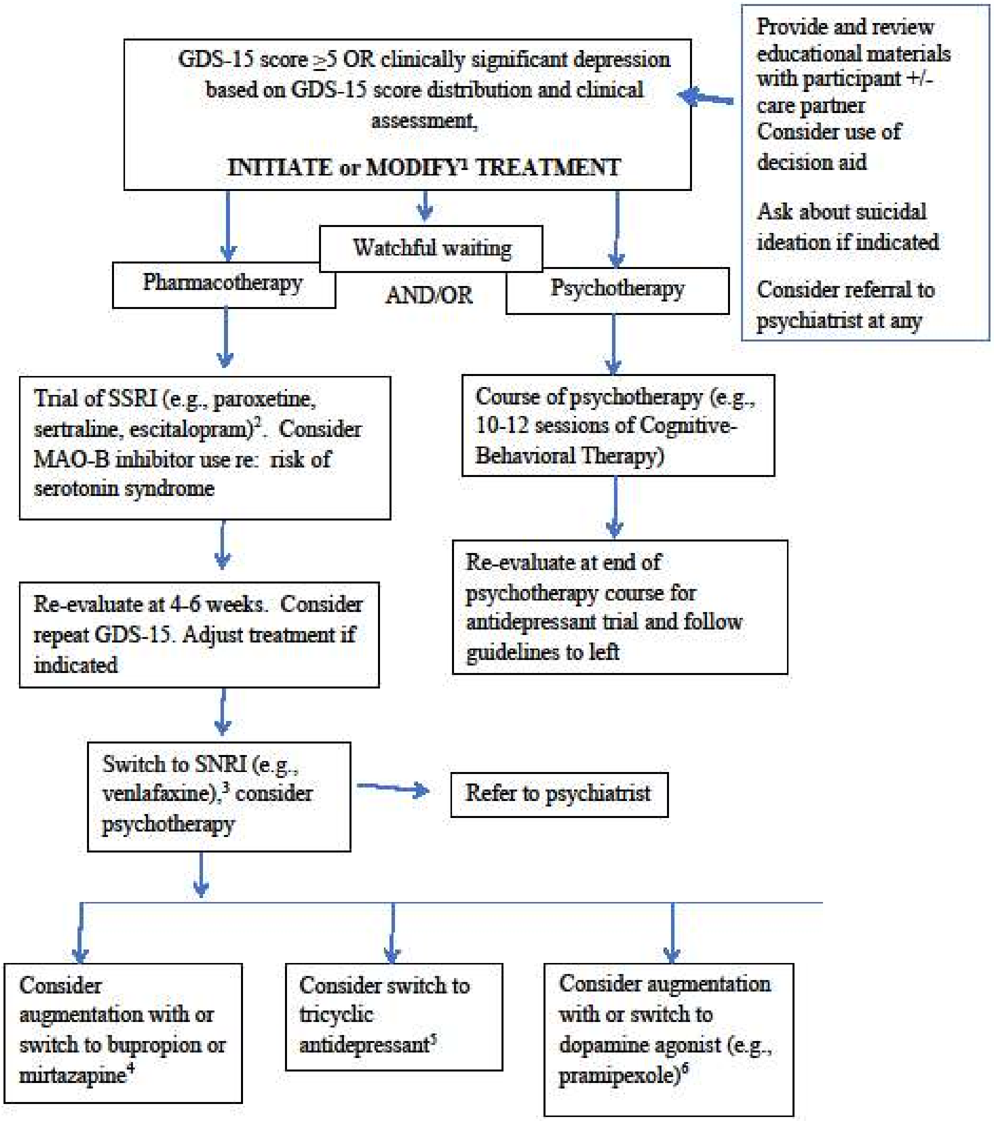

Initiation of treatment should involve shared decision making (i.e., consideration of patient preference). Consider use of shared decision-making tool. Factors also to consider are personal history of response or tolerability to previous antidepressant trials, as well as first-degree family history experience. Presence of significant cognitive impairment suggests not using antidepressants with high anticholinergic effects (e.g., tricyclic antidepressants and paroxetine). Some clinicians and patients may use genotyping to make antidepressant choices and dosing decisions based on CYP 450 activity and potential drug-drug interactions.

1. Patients who screen positive and have clinically significant depressive symptoms, but are already being treated, can join the treatment algorithm at the appropriate point based on current and past depression treatment.
2. Starting dose: sertraline (25-50 mg/day), paroxetine (10-20 mg qhs), escitalopram (10 mg/day). After 4-6 weeks consider increase in sertraline (to 50-100 mg/day, then 150 mg/day, then 200 mg/day, in 2-week periods), paroxetine (to 20-30 mg qhs, and then 40 mg qhs) or escitalopram (to 20 mg/day) depending on response AND tolerability. Total duration of trial=12 weeks.
3. Starting dose: venlafaxine (37.5-75 mg/day), duloxetine (30 mg/day). After 4-6 weeks consider increase in venlafaxine (to 75-150 mg/day, then 225 mg/day, then 300 mg/day, in 2-week periods) or duloxetine (to 40 mg/day, then 60 mg/day) depending on response AND tolerability. Total duration of trial=12 weeks.
4. Starting dose: bupropion 75 mg/day. After 2 weeks increase to 75 mg bid. After 4-6 weeks total consider increase to total 75 mg tid, then 150 mg bid, depending on response AND tolerability. Total duration of trial=12 weeks. Bupropion XL can also be considered, starting at 150 qd for 2 weeks, then increasing to 300 mg qd. Starting dose: mirtazapine 7.5-15 mg qhs. After 4-6 weeks consider increase to 15-30 mg qhs, depending on response AND tolerability. Augmentation if partial response to existing antidepressant, switch if no response.
5. Starting dose: nortriptyline 25 mg qhs. After 2 weeks increase to 50 mg qhs. After 4-6 weeks total consider increase to 75 mg qhs. Therapeutic level is 50-150 ng/dl. Total duration of trial=12 weeks.

Starting dose: pramipexole 0.125 mg tid. Consider increase in dose up to maximum of 1.0 mg tid over total of 6-week period. Total duration of trial=12 weeks. Augmentation if partial response to existing antidepressant, switch if no response.

Figure S3:

Semi-structured Interview Guide

PD patients with or without a carepartner

6. How would you describe the experience of depression in Parkinson disease? How do you think it might be different from non-Parkinsonian depression?
7. What challenges have you faced in discussing depression with your health care providers?
8. In what ways has participating in this screening program changed your clinical care?
9. How did you feel about completing the screening questionnaire for depression?
10. What would you tell a person with PD about depression? What would you tell a doctor/nurse caring for a person with PD?
11. (care-partners) What has been your experience as the care partner of someone with PD-related depression? How has this impacted your relationship?

Health Care Providers

Interview to complement retrospective chart review

1. How would you usually screen patients for depression in your clinic?
   - For a patient with early PD with minimal motor symptoms
   - For a patient with late stage PD with complex motor and non-motor issues?
2. How do you usually document depression screening in the patient's medical record?
3. What do you see as the barriers to depression screening in your clinic?

Interview to assess depression screening acceptability, barriers and challenges

1. How has participating in this screening program changed your clinical workflow?
2. What unexpected challenges did you/your site face implementing the screening protocol?
3. Do you intend to continue using the protocol after the end of the trial?

Figure S4:

Feasibility and acceptability questionnaires of systematic depression screening and barriers to screening

Clinician questionnaire

	1=strongly disagree	2	3=neutral, neither agree nor disagree	4	5=strongly agree
This process was easily integrated into workflow					
The GDS-15 helped in my clinical assessment of the patient					
The treatment recommendations were helpful					
I understood what action I should take next					
I would recommend this process to other centers					

Comments: please comment on any challenges integrating the GDS-15 into your workflow and any suggestions you have for improvement of the process or treatment recommendations.

Patients/carepartner questionnaire

	1=strongly disagree	2	3=neutral, neither agree nor disagree	4	5=strongly agree
I felt comfortable completing the questions about depression					
I felt comfortable expressing my thoughts and feelings with the clinical team					
Compared to before, I have a better understanding of my treatment options for depression in PD					
The process improved my trust in the medical team					
I would recommend this process to other centers					

Comments: if you would not recommend this process to other centers please indicate why...

Patient/carepartner questions are based on but modified slightly from https://doi.org/10.1016/j.jpainsymman.2020.06.001.

Figure S5 : Patient facing educational materials

LIVING WITH PD

Combatting Depression and Parkinson's Disease

Depression is one of the major, and most common, challenges for people living with Parkinson's disease (PD). Everyone feels sad from time to time and it is normal to experience stress when faced with a difficult disease such as PD. However, sadness can become a significant problem if it manifests into clinical depression and is left untreated.

We have only recently begun to recognize how common PD-related depression is and its impact on daily life. The Parkinson's Outcomes Project, the largest clinical study of PD through the Parkinson's Foundation Centers of Excellence, found that taken together, mood, depression and anxiety have the greatest impact on health status — even more than the motor impairments commonly associated with the disease. Fortunately, previous studies have also shown that treating depression is one of the most significant ways to improve quality of life.

It is especially important for people with PD to discuss even subtle changes in mood with their doctor as soon as they arise because, for many doctors, diagnosing depression can be difficult to differentiate from symptoms of Parkinson's — such as a masked facial expression, sleep problems and fatigue — which overlap with symptoms of depression.

Sadness Versus Depression

While sadness is temporary, depression is persistent, and the people who experience it find that they cannot enjoy life as they used to. At least 40 percent of people with PD experience clinical depression at some time during the disease. It may occur early or late in the course of the disease and the person who is depressed may find that some days are better than others.

10 signs of Depression in Parkinson's

1. **Excessive worrying**
2. **Persistent sadness**
3. **Crying**
4. **Loss of interest in usual activities and hobbies**
5. **Increased fatigue and lack of energy**
6. **Feelings of guilt**
7. **Loss of motivation**
8. **Complaints of aches and pains**
9. **Feelings of being a burden to loved ones**
10. **Ruminations about disability, death and dying**

People with these symptoms should discuss them with a doctor.

Depression causes personal suffering and also appears to intensify problems with mobility and memory. A person with PD, or his or her care partner or physician, may at first dismiss the signs of depression because they assume that it is normal to be depressed when faced with this illness. This can lead to feelings of helplessness and confusion, which may make the problem worse.

Causes of Depression in PD

There is no clear cause of depression but most specialists agree that it is probably a combination of factors. Research suggests that experiencing depression early in the disease may be directly due to PD-related chemical changes in the brain. Parkinson's causes changes in areas of the brain that produce serotonin, norepinephrine and dopamine — chemicals that are involved in regulating mood, energy, motivation, appetite and sleep. In addition, the frontal lobe of the brain, which is important in controlling mood, is known to be underactive in PD.

It is very important to address depression because it can affect other symptoms and quality of life. If you are concerned that you or a loved one may be depressed, discuss symptoms with your doctor. There are several ways to treat clinical depression. It is important to find the method that works best for you.

TIP

Because PD Depression is so common, the Parkinson's Foundation recommends that all people with PD:

- ✓ **Get screened for depression at least once a year.**
- ✓ **Discuss all changes in mood with their healthcare professional and doctor.**
- ✓ **Bring a family member to doctor's appointments to discuss changes in their mood.**

Treating PD Depression

People with PD who experience uncontrolled "on-off" periods and freezing episodes are more prone to depression, so speak with your doctor about the best approach to controlling these symptoms first. The same is true of some other, non-motor symptoms of PD — for example, poor sleep, constipation and fatigue — that need to be treated to decrease the burden of living with the disease.

Regular exercise can help treat the symptoms of depression and PD. Eating a healthy diet is another approach that can help your overall wellness. Staying involved in social and recreational activities is also important. Have something to look forward to, whether a hobby or socializing with friends and family.

Receiving help from professionals and peers can help you learn to cope with stress, improve social relationships and find solutions to practical day-to-day impairments. The Parkinson's Outcome Project found that rates for depression were lowest among patients receiving care from clinics with the most active approach to counseling. Cognitive Behavioral Therapy, a type of counseling, has been shown to be especially effective in people with PD.

Lastly, know that there are many medications available for depression in PD. Studies have suggested that one class of antidepressants, called "dual reuptake inhibitors," which affect both serotonin and norepinephrine, improve depressive symptoms in people with PD.

The pharmacological treatment of depression in PD needs to be individualized and may involve a variety of strategies. Make sure your doctor knows that Asendin® (amoxopine) is not an appropriate antidepressant for people with PD. If you or your loved one is currently taking an antidepressant that does not appear to be helping, ask your doctor if a different agent may work better.

Learn more about PD-related depression and other mental health symptoms by requesting a copy of our book Mood: A Mind Guide to Parkinson's at **Parkinson.org/Books**. You can also request more information by calling **1-800-4PD-INFO (473-4636)**.

Learn more about medications that should never be given to people with PD and our Aware in Care campaign at AwareInCare.org.

Depression and PD: A Non-Drug Treatment Option

It is estimated that at least 50 percent of people living with Parkinson's Disease (PD) experience depression at some time during the course of their disease. The Parkinson's Foundation Parkinson's Outcomes Project found that together, mood, depression and anxiety have the greatest impact on health status, even more than the motor impairments commonly associated with the disease. The persistent sadness and hopelessness that accompanies depression make the challenges of living with PD even greater. The good news is that depression is a Parkinson's symptoms that can be controlled. No one chooses Parkinson's, but you can choose how to cope with it.

Learn the skills that will empower you take control of your mood, worry less and find meaning in daily life. These skills are modeled on a non-drug therapy called cognitive behavioral therapy, which has been proven helpful for Parkinson's-related depression. To cope with depression, you can put the skills you'll learn in therapy into action.

The following article is based on the latest research and a Parkinson's Foundation Expert Briefing about depression, hosted by Roseanne D. Dobkin, PhD, from Rutgers, The State University of New Jersey, Robert Wood Johnson Medical School.

What Is Cognitive Behavioral Therapy?

Cognitive behavioral therapy (CBT) is a non-drug approach to developing the skills and actions that change patterns of thought and behavior related to depression.

Many factors can cause a person to become and stay depressed. How we think about things and interpret what goes on around us influences how we feel. So does our behavior—what we do, or don't do, in response to the stresses of life. Depression can have a biological cause. Brain changes that underlie PD are also thought to contribute to depression. But biology is not the only cause. Learning how to consciously change your thoughts and behaviors can help treat your depression.

Focus on Goals to Change Behavior

The first step is to make plans and set goals for activities. Emotions can take control when we feel depressed or anxious. Instead, let your behavior—your activities—guide you. Think strategically about increasing your involvement in meaningful activities—avoiding being busy for the sake of it. Goals should be small and realistic.

Focus on these three areas when setting goals:

- **Exercise**. Identify a reasonable daily exercise goal, whether it's walking for 15 minutes, doing Tai Chi or seated exercises, or going to an exercise class. Ask for guidance from a physical or occupational therapist.
- **Socialize**. Keep socializing goals small and do-able. Don't jump in to hosting a dinner party—it could be as simple as answering the phone or saying hello to a cashier.
- **Self-soothe**. Take time every day for an activity that will lead to a positive emotion—something that just feels good. For instance, relax with a cup of hot tea, soak in the bath or listen to music.

While planning activities that guide your day, consider these questions:

- Are there things you used to love to do that fell off the radar with your PD diagnosis? Consider re-introducing those activities.

therapy is that it is self-reinforcing. Set small, specific goals and let the goal guide your behavior, no matter how you feel. When you feel a glimmer of success, your enthusiasm to do more will kick in. A small change in activity can improve a person's mood. A better outlook can inspire more activity, and a more objective assessment of the future.

Conclusion: Don't Suffer in Silence

Your mood is a critical aspect of living with PD that you can control. Talk to your friends, family and doctor if you feel persistently sad or hopeless. If symptoms are severe, you and your medical team might consider one of the many antidepressant medications. But effective, non-drug treatments also are available, both in combination with drug therapies and on their own. If you are depressed, speak up and seek help.

For more information on depression, anxiety and treatment, read the Parkinson's Foundation book, Mood: A Mind Guide to Parkinson's Disease or call the foundation's free Helpline at 1-800-4PD-INFO (473-4636) to speak with a Parkinson's specialist.

TIP

Tips for Taming Anxiety

Many people with PD experience both anxiety and depression. Visit Parkinson.org to learn more about anxiety and ways to treat it. Try some of these non-conventional techniques:

- ✓ **Breathing exercises**
- ✓ **Massage therapy**
- ✓ **Music therapy**
- ✓ **Guided imagery**
- ✓ **Meditation**

CBT: How to Find a Therapist

- Ask your doctor or neurologist for a referral
- Ask support group members for recommendations
- Call the Parkinson's Foundation's free Helpline at 1-800-4PD-INFO (473-4636)

TIP

Tips for Better Sleep to Help Ward Off Anxiety and Depression

- ✓ **Go to bed and get up at around the same time every day.**
- ✓ **Use the bed for sleep only.**
- ✓ **Limit daytime naps.**
- ✓ **Don't lie in bed unable to sleep for long periods — get up and do something else until you feel tired, then try to sleep again.**
- ✓ **Limit caffeine and alcohol in the evening.**
- ✓ **To learn more about Parkinson's and Sleep read the Parkinson's Foundation book, Sleep: A Mind Guide to Parkinson's Disease or call the foundation's free Helpline at 1-800-4PD-INFO (473-4636) to speak with a Parkinson's specialist.**

Roseanne D. Dobkin, Ph.D., Associate Professor, Psychiatry, Rutgers, The State University of New Jersey, Robert Wood Johnson Medical School

Figure S6: Screen percentage by dates of each center

Supplementary Figure. Screen percentage by date of Vanderbilt center

Total Patients Screened: 185
Total Eligible : 80
Total Completed enrolled: 31

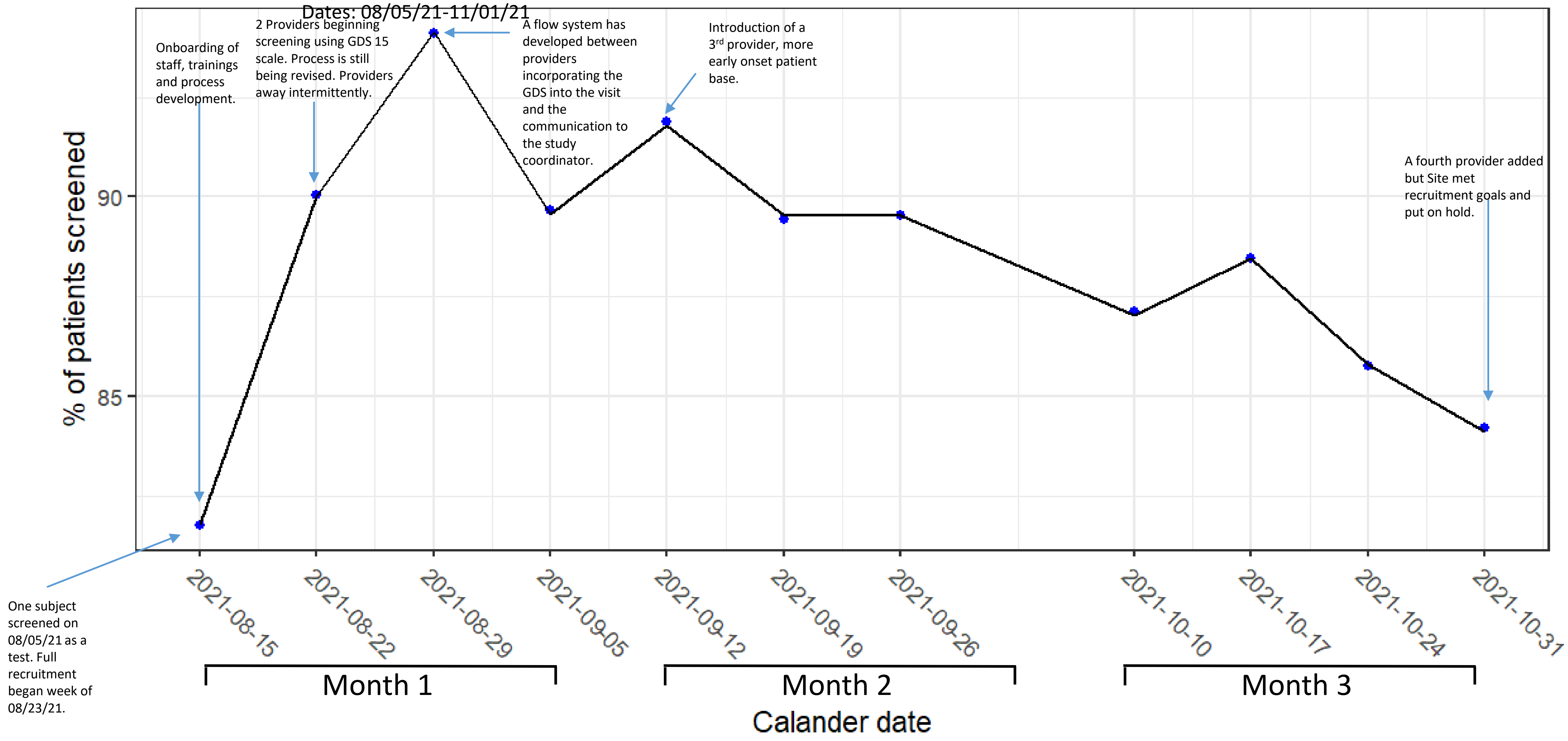

6b. Supplementary Figure. Screen percentage by date of UPenn center.

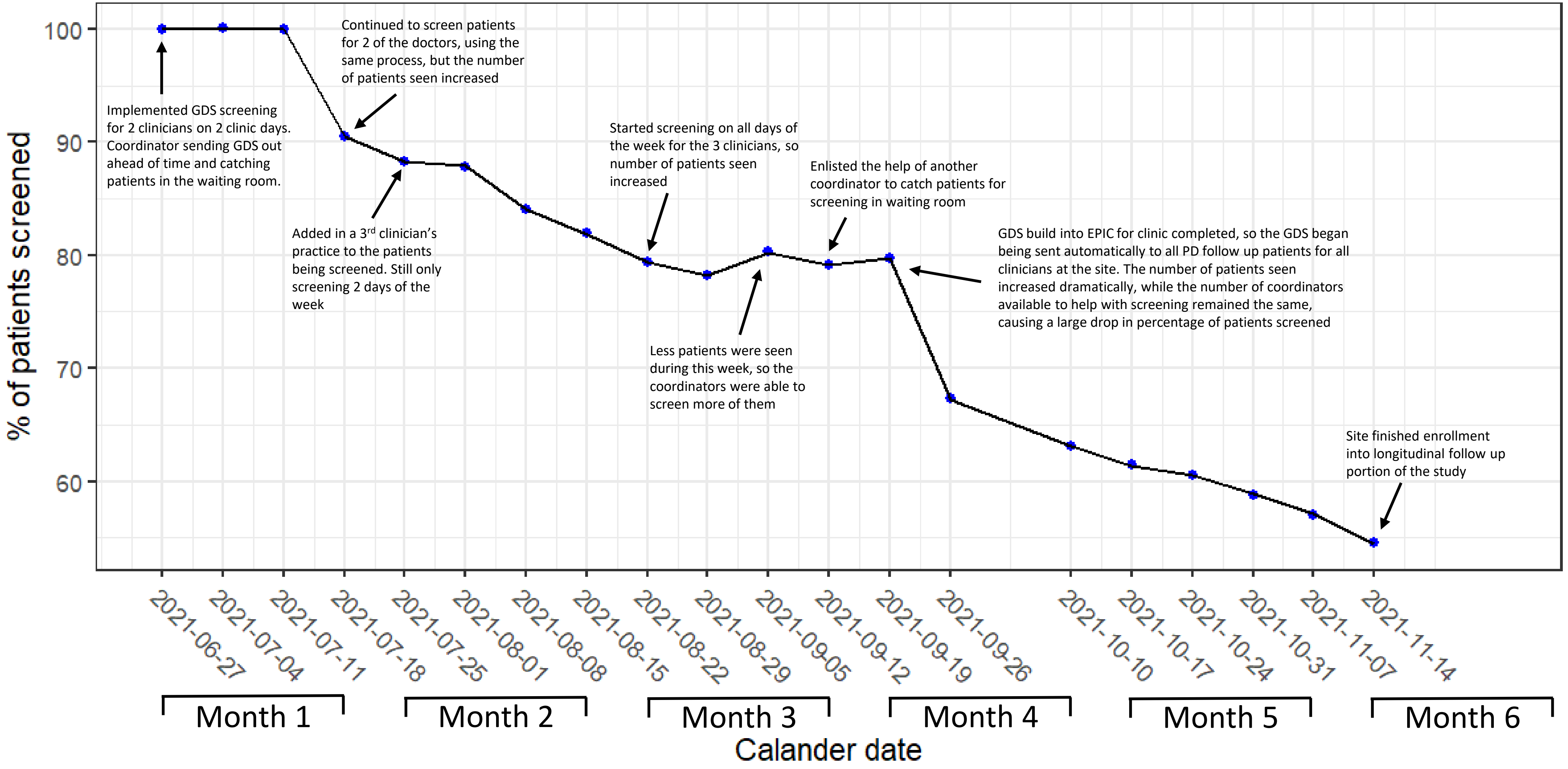

6c. Figure. Screen percentage by date of John Hopkins center.

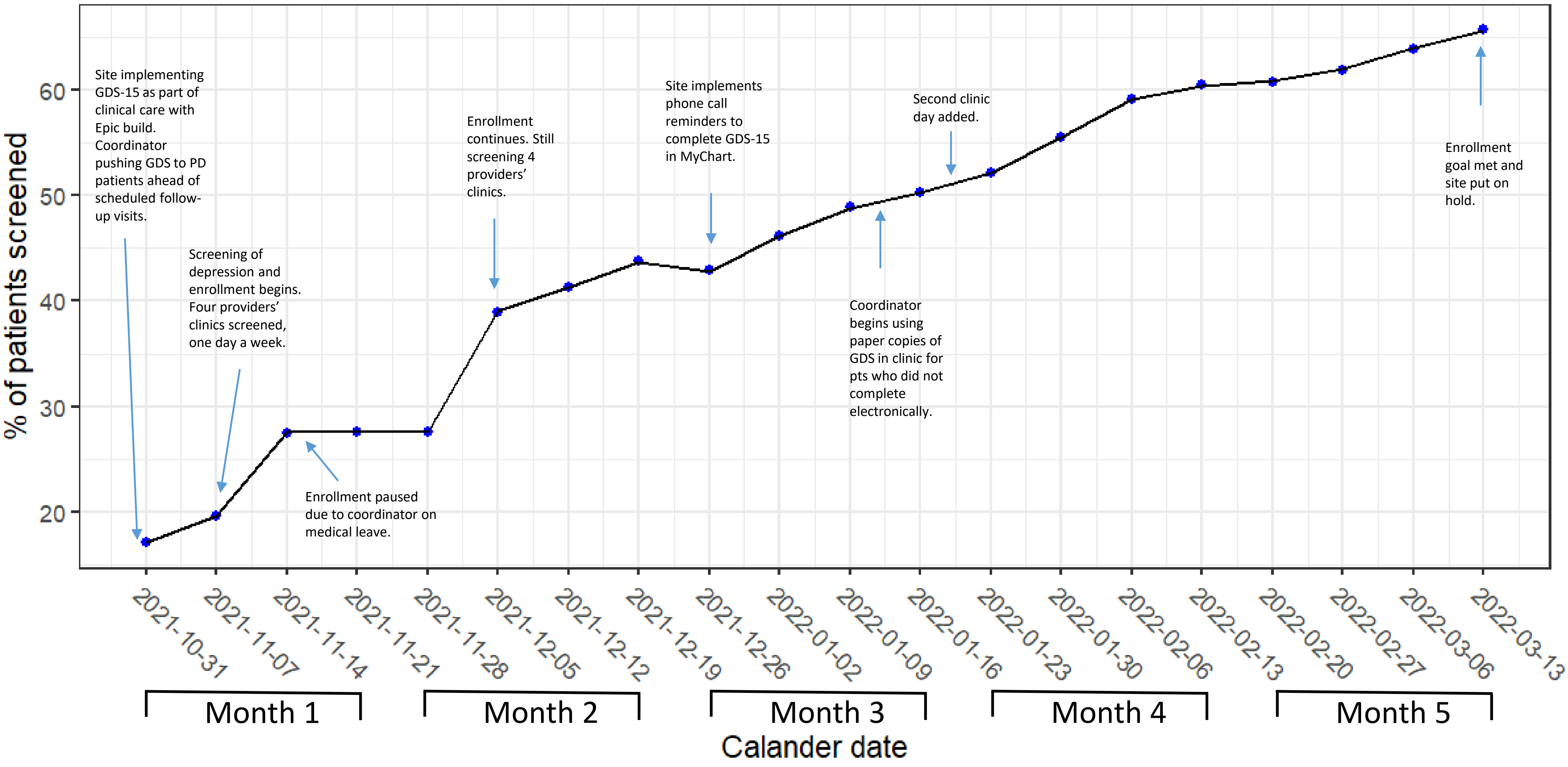

6d. Figure. Screen percentage by date of Toronto center.

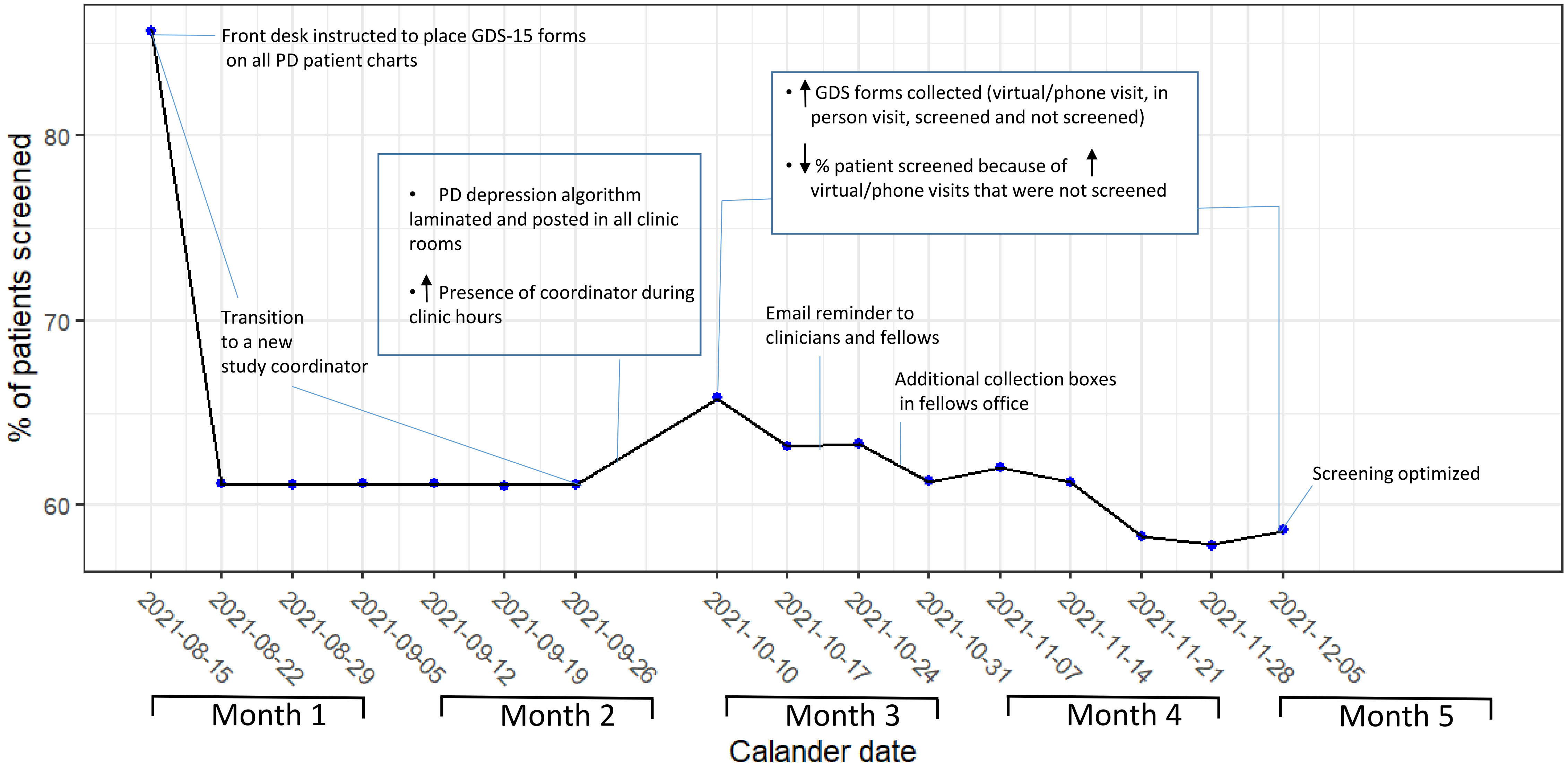

6e. Supplementary figure. Screen percentage by date of Alberta center.

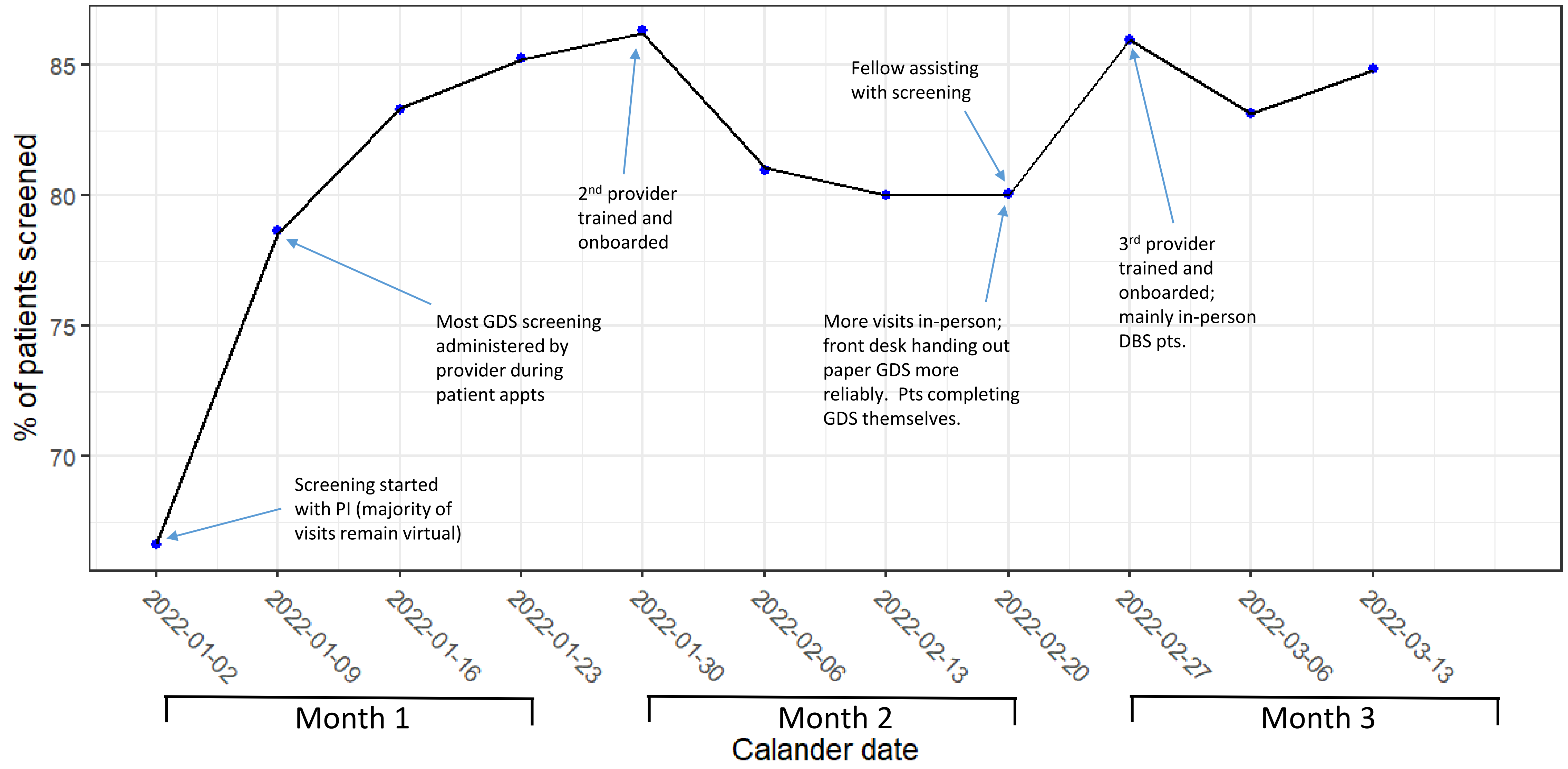

Supplement: Supplementary file 1 — Figure S1. Process maps for Toronto Western Hospital (lead site). Process maps for the depression screening workflow developed for the Toronto Western Hospital. A similar map was developed by each of the sites based on this example but tailored to their own clinic structure and processes. Figure S2. Treatment algorithm and suggestions. Suggested treatment algorithm and treatment suggestions provided to all sites. Figure S3. Semistructured interview guides. Interview guide for interviews with patients and health‐care providers. Figure S4. Feasibility and acceptability questionnaires of systematic depression screening and barriers to screening. Feasibility and acceptability questionnaires administered to health‐care providers and prospectively followed patients. Figure S5. Patient‐facing educational materials. Patient‐facing educational materials provided to sites, for use at their discretion. Figure S6. Screen percentage by dates of each center. Charts of percentage of PD (Parkinson's disease) patients screened over the implementation period used to monitor screening rates and process changes at each site. (A) Vanderbilt University, (B) University of Pennsylvania, (C) Johns Hopkins University, (D) Toronto Western Hospital, and (E) University of Alberta. [file MDC3-11-1212-s001.pdf]
